# An orphan viral genome with unclear evolutionary status sheds light on a distinct lineage of flavi-like viruses infecting plants

**DOI:** 10.1093/ve/veaf001

**Published:** 2025-01-03

**Authors:** Zhongtian Xu, Luping Zheng, Fangluan Gao, Yiyuan Li, Zongtao Sun, Jianping Chen, Chuanxi Zhang, Junmin Li, Xifeng Wang

**Affiliations:** State Key Laboratory for Managing Biotic and Chemical Threats to the Quality and Safety of Agro-products, Key Laboratory of Biotechnology in Plant Protection of Ministry of Agriculture and Zhejiang Province, Institute of Plant Virology, Ningbo University, Ningbo 315211, China; Institute of Plant Virology, College of Plant Protection, Fujian Agriculture and Forestry University, Fuzhou 350002, China; Institute of Plant Virology, College of Plant Protection, Fujian Agriculture and Forestry University, Fuzhou 350002, China; State Key Laboratory for Managing Biotic and Chemical Threats to the Quality and Safety of Agro-products, Key Laboratory of Biotechnology in Plant Protection of Ministry of Agriculture and Zhejiang Province, Institute of Plant Virology, Ningbo University, Ningbo 315211, China; State Key Laboratory for Managing Biotic and Chemical Threats to the Quality and Safety of Agro-products, Key Laboratory of Biotechnology in Plant Protection of Ministry of Agriculture and Zhejiang Province, Institute of Plant Virology, Ningbo University, Ningbo 315211, China; State Key Laboratory for Managing Biotic and Chemical Threats to the Quality and Safety of Agro-products, Key Laboratory of Biotechnology in Plant Protection of Ministry of Agriculture and Zhejiang Province, Institute of Plant Virology, Ningbo University, Ningbo 315211, China; State Key Laboratory for Managing Biotic and Chemical Threats to the Quality and Safety of Agro-products, Key Laboratory of Biotechnology in Plant Protection of Ministry of Agriculture and Zhejiang Province, Institute of Plant Virology, Ningbo University, Ningbo 315211, China; State Key Laboratory for Managing Biotic and Chemical Threats to the Quality and Safety of Agro-products, Key Laboratory of Biotechnology in Plant Protection of Ministry of Agriculture and Zhejiang Province, Institute of Plant Virology, Ningbo University, Ningbo 315211, China; Institute of Western Agriculture, Chinese Academy of Agricultural Sciences, Changji 831100, China

**Keywords:** Snake River Alfalfa virus, flaviviridae, endornaviridae, flavi-like virus

## Abstract

Advancements in high-throughput sequencing and associated bioinformatics methods have significantly expanded the RNA virus repertoire, including novel viruses with highly divergent genomes encoding “orphan” proteins that apparently lack homologous sequences. This absence of homologs in routine sequence similarity search complicates their taxonomic classification and raises a fundamental question: Do these orphan viral genomes represent *bona ide* viruses? In 2022, an orphan viral genome encoding a large polyprotein was identified in alfalfa (*Medicago sativa*) and thrips (*Frankliniella occidentalis*), and named Snake River alfalfa virus (SRAV). SRAV was initially proposed as an uncommon flavi-like virus identified in a plant host distantly related to family *Flaviviridae*. Subsequently, another research group showed its common occurrence in alfalfa but challenged its taxonomic position, suggesting it belongs to the family *Endornaviridae*. In this study, a large-scale analysis of 77 publicly available small RNA datasets indicates that SRAV could be detected across various tissues and cultivars of alfalfa, and has a broad geographical distribution. Moreover, profiles of the SRAV-derived small interfering RNAs (vsiRNAs) exhibited typical characteristics of viruses in plant hosts. The evolutionary analysis suggests that SRAV represents a unique class of plant-hosted flavi-like viruses with an unusual genome organization and evolutionary status, distinct from previously identified flavi-like viruses documented to infect plants. The latter shows a close evolutionary relationship to flavi-like viruses primarily found in plant-feeding invertebrates and lacks evidence of triggering host RNA interference (RNAi) responses so far. Moreover, mining the transcriptome shotgun assembly (TSA) database identified two novel viral sequences with a similar genome organization and evolutionary status to SRAV. In summary, our study resolves the disagreement regarding the taxonomic status of SRAV and suggests the potential existence of two distinct clades of plant-hosted flavi-like viruses with independent evolutionary origins. Furthermore, our research provides the first evidence of plant-hosted flavi-like viruses triggering the host’s RNAi antiviral response. The widespread occurrence of SRAV underscores its potential ecological significance in alfalfa, a crop of substantial economic importance.

## Introduction

Continuous advancements in high-throughput sequencing (HTS) technologies and bioinformatics methodologies have greatly facilitated the identification of divergent viral sequences that may have been previously overlooked (Charon et al. 2022, Guo et al. 2024). As a consequence, some divergent viral contigs encoding “orphan” proteins have been identified and documented (Kuchibhatla et al. 2014). Those divergent viral contigs, presumed to represent divergent viral entities, often pose challenges in their classification within the virus taxonomy lineage, given the lack of sequence similarity homologs. Moreover, several issues warrant careful consideration before interpreting the results from metagenome or metatranscriptome datasets (Togoobat et al. 2023). Do these divergent viral contigs truly represent viruses, or are they instead host genetic sequences harboring endogenous viral elements (EVEs)? Additionally, determining the *bona fide* host of a virus, particularly those viruses identified solely through *in silico* metatranscriptomic or metagenomic analysis, remains an open question.

HTS technologies have not only led to the discovery of numerous new viral sequences but have also provided new insights into virus–host relationships. Taking flavivirids (family *Flaviviridae*) as an example, typical flavivirids within the family *Flaviviridae* are characterized as monopartite, single-stranded, positive-sense RNA viruses between 9 and 13 kilobases (kb) in length that code a large polyprotein (Blitvich and Firth 2015, Parry et al. 2019). Flavivirids are primarily transmitted by arthropods and can cause severe illnesses in vertebrates, including humans, on a global scale (Pierson and Diamond 2020, Bamford et al. 2022). Most recognized flaviviruses in the family *Flaviviridae* are transmitted horizontally between arthropods and vertebrate hosts, categorizing them as dual-host viruses. However, not all flaviviruses rotate between arthropods and vertebrates; some are specific to vertebrate hosts, while others seem to be restricted to insects (Blitvich and Firth 2015).

Our knowledge about the host range of flavivirids has been continuously improved due to the widespread usage of HTS technologies. Metatranscriptomic studies have revealed that flavivirids can also be found in a variety of aquatic invertebrates, including sharks, crabs, giant freshwater prawns, and several other species (Parry et al. 2019, Dong et al. 2021). In addition, data mining of publicly available transcriptome datasets revealed that the host range of *Pestivirus* (a genus in the family *Flaviviridae*) includes amphibians, reptiles, and ray-finned fish (Mifsud et al. 2022). A research study published in 2022 revealed the presence of an orphan viral genome in alfalfa, which possesses unusual genome organization and was named Snake River Alfalfa virus (SRAV). The authors proposed that SRAV is the first flavi-like virus identified in a plant host (Dahan et al. 2022). However, this viewpoint was later challenged. A study published in 2023 proposed that SRAV should be classified as a member of the family *Endornaviridae* (Postnikova et al. 2023). Moreover, some flavi-like viral sequences encoding a larger polyprotein and possessing longer genomes (up to 23 kb in length) compared to classical flavivirids have also been identified in plants (Atsumi et al. 2013, Schönegger et al. 2022, Debat and Bejerman 2023). These flavi-like viruses have distinct genome organizations from classical flavivirids but group within the family *Flaviviridae* in phylogenetic analysis based on the RNA-dependent RNA polymerase (RdRp) domain (Paraskevopoulou et al. 2021). Additionally, these flavi-like viruses with longer genomes identified in plants exhibited a close phylogenetic relationship to those flavi-like viruses mainly found in plant-feeding invertebrates (Dolja et al. 2020). However, so far, there is no research support that these flavi-like viruses with longer genomes, documented to infect plants, can activate the host’s RNAi antiviral response. This raises doubts whether these viruses are truly plant viruses, despite some studies showing that a flavi-like virus (Gentian Kobu-sho-associated virus GKaV) is very widely detected among gentians and other plants (Atsumi et al. 2013, Atsumi, et al., 2023, Shaffer et al. 2022).

RNA interference (RNAi) is a conserved antiviral mechanism across eukaryotes (Ding 2010, 2023). In the antiviral RNAi process, virus infection induces Dicer to process virus-specific double-stranded RNA into small interfering RNAs (siRNAs) (Ding 2010, 2023). During this process, the virus-derived small interfering RNAs (vsiRNAs) will be enriched in the hosts, which can be easily detected by deep sequencing (Zheng et al. 2017). Hence, through deep sequencing and bioinformatic analysis of small RNA populations from infected plants, both RNA and DNA viruses, along with viral satellites and viroids, can be identified and their genomes partially or fully reconstructed (Kreuze et al. 2009, Wu et al. 2010, Zheng et al. 2017, Pooggin 2018). Previous research has demonstrated that insect-borne viruses exhibit distinct length distribution patterns in their planthopper vectors compared to rice hosts. This suggests that the vsiRNAs profile can provide insights into the origins of these vsiRNAs (Yang et al. 2018). Studies have shown that vsiRNA profiles differ between those derived from EVEs and episomal viruses. Therefore, analyzing the vsiRNA profile corresponding to a viral contig can provide clues as to whether the viral contig represents an episomal virus (Pooggin 2018).

In the present study, we performed a large-scale analysis of 77 publicly accessible small RNA datasets from alfalfa, suggesting that SRAV represents a true episomal virus capable of triggering the plant host’s RNAi antiviral defense. Subsequent evolutionary analysis revealed that SRAV is a flavi-like virus infecting alfalfa, rather than a member of the family *Endornaviridae*. In addition, we identified two new flavi-like viruses by mining the public transcriptome shotgun assembly (TSA) database that closely resemble SRAV in genome structure and evolutionary status. In brief, our analysis suggests the potential existence of two distinct types of plant-hosted flavi-like viruses with different evolutionary origins. Additionally, it provides the first RNAi evidence that plants can serve as hosts for flavi-like viruses.

## 2. Methods

### 2.1 Homology search and Conserved RdRp motif identification

The ORF Finder online server (https://www.ncbi.nlm.nih.gov/orffinder/) was used to predict the open reading frame (ORF) of the SRAV viral genome (Accession: ON669064.1). The conserved domains of SRAV polyprotein (Accession: USJ75181.1) were predicted using two methods, NCBI CD-search (https://www.ncbi.nlm.nih.gov/Structure/cdd/wrpsb.cgi) and InterProScan (https://www.ebi.ac.uk/interpro/). In addition, to identify as many functional domains as possible, HHpred was selected to predict the potential functional domains. HHpred employs a profile-against-profile search method, enabling the detection of distant protein sequence similarities (Söding et al. 2005).

To rule out the possibility that SRAV is merely alfalfa gene carrying the EVE (refer to RdRp), we first compared SRAV with the alfalfa genome (Long et al. 2022) using BLASTn and BLASTx (Altschul et al. 1990) with default parameters.

To investigate whether the SRAV, as an orphan viral genome, harbors the conserved motif in the palm subdomain of RdRp, Geneious software (Kearse et al. 2012) was used to identify and curate the conserved RdRp canonical A, B, and C motifs in the result obtained from multiple sequence alignment (MSA). MSA was carried out using Multiple Alignment using Fast Fourier Transform (MAFFT), and alignment visualization was achieved using the R package ggmsa (Zhou et al. 2022). viruses.

### 2.2 Mining the public TSA database to identify potential viral sequences resemble SRAV

The polyprotein sequence of SRAV (accession: USJ75181.1) was used as the query sequence in TSA database using online tBLASTn with default parameters (organism: *Viridiplantae*). We manually reviewed the results, selecting sequences that encode polyproteins reaching a specific length (e.g. 2500 amino acids) and contain the RdRp domain. We then analyzed the original datasets from which these viral sequences were assembled to double-check the sequence accuracy. In short, the downloaded raw RNA-Seq dataset was processed with fastp (Chen et al. 2018) to trim potential adapter sequences and filter out low-quality regions. Reads shorter than 36 nucleotides were discarded. The remaining clean reads were then assembled *de novo* using the Trinity assembler (version 2.15.1) (Haas et al. 2013). The comparison between the viral sequences downloaded from the TSA database and the newly assembled sequences was performed using online BLASTn, and the newly assembled viral sequences were used in further analysis.

### 2.3 Sequence Read Archive datasets collection, processing, and virome analysis

The accession numbers of alfalfa-related small RNA datasets were collected through a comprehensive literature review of research studies involving small RNA sequencing in alfalfa. We first checked the downloaded datasets for small RNA sequencing adapter sequences. If adapter sequences were present, cutadapt (v1.16) (Martin 2011) was used to remove the adapter sequences. Reads with lengths between 18 nt and 30 nt that contained no ambiguous nucleotides (N) were retained as clean reads. The clean reads were then subjected to virome analysis using VirusDetect (Zheng et al. 2017).

### 2.4 SRAV occurrence analysis and vsiRNAs profile characterization

The clean reads were clustered into unique reads using an in-house Python script. Next, the remaining small RNA clean reads were aligned to the SRAV virus reference genome (accession number: ON669064.1) using Bowtie (Langmead et al. 2009), allowing for up to one mismatch. We then analyzed the mapped small RNAs to explore the profile of vsiRNAs, including their length distribution, 5ʹ terminal nucleotide preference, genome distribution, and polarity.

The average sequencing depth and coverage breadth were calculated as detailed previously (Togoobat et al. 2023). To reduce false positives caused by the random alignment of short fragments, we only considered SRAV in the sample if the vsiRNAs mapped to the SRAV genome met the following criteria: an average sequencing depth of at least 4X and viral genome coverage of more than 60%. Under these conditions, the presence of SRAV was considered confident.

### 2.5 Phylogenetic analysis

Phylogenetic analyses were conducted using MSAs of either the RdRp domain region or the whole polyprotein sequence across various viral groups. The polyprotein sequences of representative members from different viral groups were retrieved from the NCBI GenBank database. The RdRp domain regions were predicted using a CD-search web server, and the corresponding RdRp domain sequences were extracted based on their positions within the polyprotein or ORF encoding RdRp.

The alignment of RdRp sequences or polyprotein sequences was achieved by MAFFT (Katoh and Standley 2013). The obtained multiple sequence alignments were subjected to trimAl to conduct automated alignment trimming (Capella-Gutiérrez et al. 2009). The trimmed MSA files were then subjected to IQ-TREE (v1.6.6) (Nguyen et al. 2015) for phylogenetic tree construction through the maximum likelihood method. The best-fitted model selection was performed by ModelFinder implemented in IQ-TREE (Kalyaanamoorthy et al. 2017), and the confidence in the topology was assessed using 5000 ultra-fast bootstrap replicates and the SH-aLRT test with 1000 replicates (Guindon et al. 2010).

The sequences of all the RdRp domain regions or polyproteins sequences with accession numbers, coupled with best-fitted model for each tree, can be found in Supplementary Table S1.

## 3. Results

### 3.1 Identification of viruses similar to SRAV in the transcriptome shotgun assembly database

**Figure 1.**
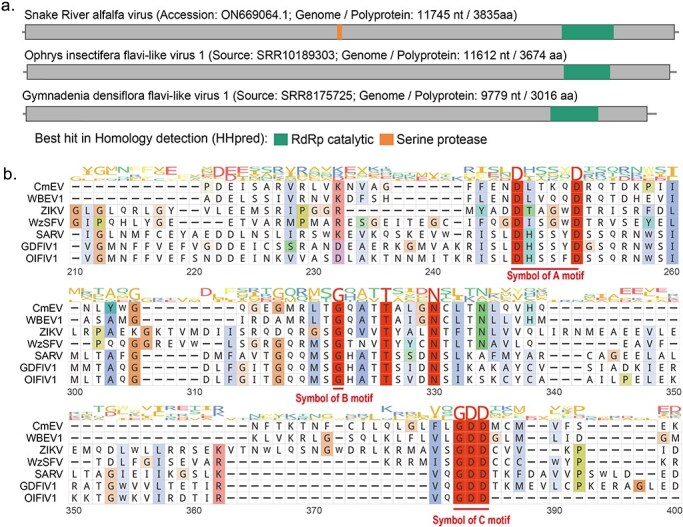
Genome organization of SRAV and SRAV-like viruses, along with a multiple alignment of the conserved RdRp palm subdomain across SRAV and other eukaryotic viruses. (a) Schematic representation of the functional domain predicted by HHpred on SRAV. The grey line in the diagram represents the virus genome. Each box represents an ORF of the virus. Conserved functional domains are color-coded, and the corresponding names are shown on the figures’ right side. (b) MSA of the conserved RdRp palm domain of SRAV and representative members of the family *Flaviviridae* and *Endornaviridae*. Abbreviations of virus names: CmEV, Cucumis melo endornavirus; WBEV1 winged bean endornavirus 1; ZIKA, Zika virus; WzSFV, Wenzhou shark flavi-like virus; SRAV, Snake River alfalfa virus; GDFlV1: Gymnadenia densiflora-associated flavi-like virus 1; OIFlV1: Ophrys insectifera-associated flavi-like virus 1.

Following the approach outlined in the “Methods” section, SRAV polyprotein sequence was used for tBLASTn search against the TSA database. Through manual curation and selection of tBLASTn hits, we identified and retained two tBLASTn hits from the results: one from *Ophrys insectifera* (accession: GHWW01044949.1) and another from *Gymnadenia densiflora* (accession: GHXE01142667.1). To verify the accuracy of the sequences, we reassembled and analyzed the original datasets corresponding to them. Following reassembly and comparative analysis, we designated these viral contigs as “Ophrys insectifera flavi-like virus 1” (OIFlV1) and “Gymnadenia densiflora flavi-like virus 1” (GDFlV1), based on their sources. Interestingly, the hosts of these two new SRAV-like viruses are both specific orchid species. The genome lengths of OIFlV1 and GDFlV1 are 11 612 nt and 9779 nt, respectively. Similar to SRAV, OIFlV1 and GDFlV1 encode polyproteins of 3674 amino acids and 3016 amino acids, respectively (Fig. 1a). When we used the polyprotein sequences of OIFlV1 and GDaFlV1 to perform BLASTp searches against the NCBI NR database, the only hits were with the SRAV polyprotein sequence, showing similarities of 32.5% and 34.2%, respectively. Additionally, the BLASTp similarity between OIFlV1 and GDFlV1 was 45.1%. These findings suggest that OIFlV1 and GDaFlV1 are novel viruses closely related to SRAV (Supplementary Fig. S1d). HHpred analysis revealed that SRAV isolates could be annotated with two functional domains: the RdRp catalytic domain and the serine protease domain. In contrast, for OIFlV1 and GDFlV1, HHpred analysis identified only the RdRp catalytic domain (Fig. 1a). The annotation details of functional domain annotation, including hit information, E-value, or probability, can be found in Supplementary Fig. S1. When predicting the functional domains of the SRAV polyprotein using various tools, we found that the RdRp catalytic domain was consistently annotated by all three methods: CD-search, InterProScan, and HHpred. However, the serine protease domain was only identified through HHpred (Supplementary Fig. S1a). For OIFlV1 and GDFlV1, only the RdRp catalytic domain could be annotated by any of the methods used (Supplementary Fig. S1b, S1c).

We also analyzed the palm subdomain of the RdRp protein from SRAV, OIFlV1, GDFlV1, and representative members of the *Endornaviridae* and *Flaviviridae* families. This subdomain includes the highly conserved catalytic core of RdRp, which comprises three key motifs: A, B, and C (Fig. 1b). Although SRAV did not yield any hits beyond itself in the NCBI BLASTx search, it possesses the characteristic Motifs A, B, and C. Specifically, SRAV and the other two SRAV-like viruses exhibit Motif A (D-x(4,5)-D), Motif B (a conserved glycine), and the typical C motif (GDD triad), similar to representative members of the *Endornaviridae* and *Flaviviridae* families (Fig. 1b).

### 3.2 The widespread occurrence of SRAV isolates in alfalfa

**Figure 2.**
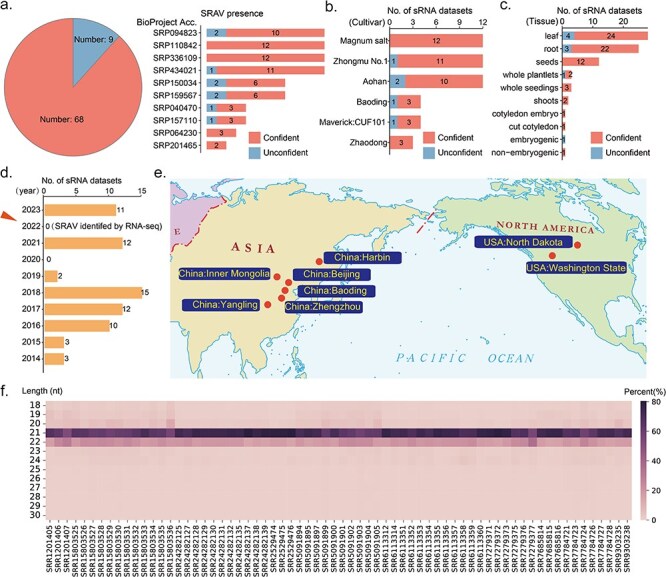
SRAV is widespread in Alfalfa and can trigger host’s RNAi antiviral response. (a) The presence of SRAV in each dataset. Confident: the presence of SRAV is reliable. Unconfident: the presence of SRAV is unreliable. (b) The number of datasets in which SRAV could be reliably detected across different alfalfa cultivars. (c) The number of datasets in which SRAV could be reliably detected across different alfalfa tissue types. (d) The number of datasets in which SRAV could be reliably detected in different years. (e) The currently known geographic distribution of SRAV. (f) The length distribution of SRAV vsiRNAs across different datasets.

To determine whether SRAV is widespread in alfalfa and capable of activating host’s antiviral responses, we conducted a large-scale analysis of all publicly available alfalfa small RNA datasets deposited in the NCBI SRA database. We also attempted to conduct similar analysis on OIFlV1 and GDFlV1, but there were no small RNA sequencing datasets of the hosts corresponding to these two viruses in the NCBI, so the analysis was only conducted on SRAV. We found that, in each sample, there were varying number of reads that could be mapped to the SRAV genome (ranging from a minimum number of 10 to a maximum of 6 370 129) (Supplementary Table S2). To avoid false positives caused by the random alignment of small RNA reads, which are typically short, we only consider the presence of SRAV as reliable in samples that pass the criteria we set: an average sequencing depth more than 4X and genome coverage more than 60%. Based on these criteria, we confidently identified the presence of SRAV in 68 out of 77 samples (Fig. 2a). Given that these 77 samples are derived from 10 different research projects (each with distinct BioProject accession numbers), the occurrence of SRAV across these independent studies can be deemed reliable. Furthermore, within four specific BioProjects (SRP110842, SRP336109, SRP064230, and SRP201465), the presence of SRAV was consistently reliable in every sample (Fig. 2a). Based on the meta-information associated with each sample, we found that SRAV can be identified across a diverse range of alfalfa cultivars, indicating its widespread presence among different genetic backgrounds (Fig. 2b). Additionally, SRAV was detected in various tissues of alfalfa, including leaves, stems, roots, seeds, shoots, nonembryogenic, cut cotyledon, and cotyledon embryo, demonstrating its broad tissue distribution within the plant hosts (Fig. 2c). Although SRAV was first identified through metatranscriptome sequencing analysis in 2022, we were able to detect the presence of the virus in small RNA samples dating back to 2014 (Fig. 2d). We also found that SRAV has a wide geographical distribution, being present across the Pacific Ocean in both Asia and North America, primarily in China and the USA (Fig. 2e). It should be noted that this does not indicate that SRAV is absent in other regions. Rather, it indicates that alfalfa from these areas has corresponding high-throughput small RNA sequencing data available for analysis. Additionally, we analyzed the length distribution of SRAV vsiRNAs in the 68 samples in which SRAV could be detected. We found that, without exception, the majority of SRAV vsiRNAs were 21 nt and 22 nt in length, with 21 nt being more abundant (Fig. 2f). This pattern is characteristic of typical plant virus vsiRNAs.

### 3.3 Host RNAi antiviral response to SRAV and other RNA viruses

**Figure 3.**
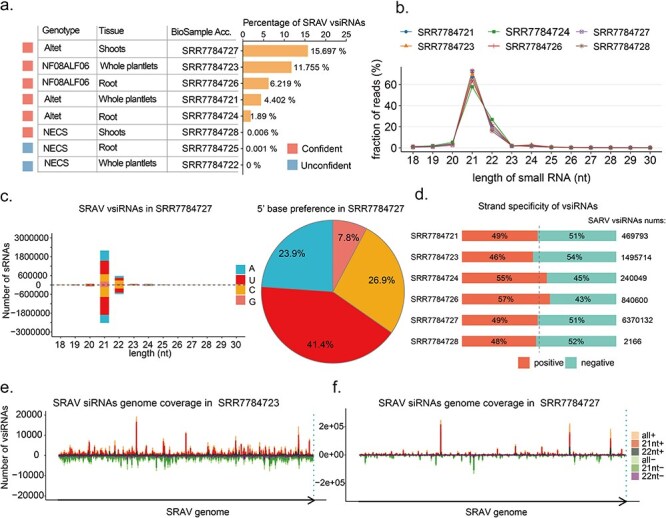
Host RNAi antiviral response to SRAV and other RNA viruses identified in BioProject SRP159567. (a) The metadata information and SRAV vsiRNAs percentage in each samples. (b) Length distribution of SRAV vsiRNAs in samples where SRAV was reliably detected. (c) Length distribution and 5ʹ termini base preference of SRAV vsiRNAs in sample SRR7784727. (d) SRAV vsiRNAs polarity in samples where SRAV was reliably detected. (e) Distribution of SRAV vsiRNAs alongside the viral genome in SRR7784723. (f) Distribution of SRAV vsiRNAs alongside the viral genome in SRR7784727.

To gain deeper insights into the interaction between SRAV and the host through the RNAi mechanism, we selected BioProject SRP159567 for a detailed analysis of vsiRNA characteristics for SRAV and other RNA viruses. We chose this BioProject because the proportion of SRAV vsiRNAs is significantly high in some samples within this BioProject (Supplementary Table S2). In the eight samples archived in BioProject SRP159567, the presence of SRAV is considered reliable in six samples. The proportion of SRAV vsiRNAs in the total small RNA population ranges from 0.006% (SRR7784728) to 15.697% (SRR7784727). These six samples, deemed to have a reliable presence of SRAV, encompass various genotypes and tissue types (Fig. 3a). The SRAV vsiRNA profiles of those six samples show a high degree of consistency, with 21 nt vsiRNAs accounting for more than 60%, while the proportion of 22 nt vsiRNAs fluctuates around 20% (Fig. 3b). When we analyzed sample SRR7784727 (Shoots sample of the Altet genotype) to study the length distribution, strand preference, and 5ʹ base preference of vsiRNAs, we found that the SRAV vsiRNAs were relatively evenly derived from both the positive and negative strands of the genome, with a 5ʹ base preference for nucleotide “U” (uracil) (Fig. 3c). Similarily, we could also observe that the “U” preference of SRAV vsiRNAs is widespread across the majority of alfalfa small RNA samples (Supplementary Fig. S2a). When analyzing the strand preference of SRAV vsiRNAs, we observed that they can originate from both the sense and antisense strands, typically in similar proportions. However, in samples SRR7784724 and SRR7784726, vsiRNAs from the sense strand exhibited a relatively higher proportion. Notably, both samples were derived from root tissue, suggesting potential variations in the vsiRNA biogenesis process across different tissues in plant hosts (Fig. 3d). Our findings revealed a relatively uniform distribution of vsiRNAs across the entire SRAV viral genome. However, certain localized regions emerged as hotspots for vsiRNA production, with 21 nt and 22 nt vsiRNAs being the predominant types within each region (Fig. 3d-e).

We conducted an in-depth viromic analysis of small RNA datasets obtained from BioProject SRP159567 using VirusDetect. Apart from identifying SRAV, as shown in Supplementary Fig. S2b, we also detected three other alfalfa viruses: Medicago sativa amalgavirus 1, Medicago sativa alphapartitivirus 1, and Medicago sativa alphapartitivirus 2, as detailed in Supplementary Fig. S2c. The length distribution patterns of SRAV vsiRNAs closely resembled those of the other three alfalfa viruses (Supplementary Fig. S2d), suggesting that SRAV is likely a episomal virus infecting alfalfa, capable of triggering the plant’s antiviral RNAi response.

### 3.4 *Flaviviridae* or *Endornaviridae*? The phylogenetic status of SRAV

After confirming that SRAV can activate the plant host’s antiviral response, we proceeded to investigate its evolutionary status. There is ongoing academic debate about whether SRAV is more likely to belong to the family *Flaviviridae* or *Endornaviridae* (Dahan et al. 2022, Postnikova et al. 2023). When using HHpred to analyze SRAV and the two other SRAV-like virus sequences, we found that, in all cases, the best matches for the annotated functional domains were linked to members of the family *Flaviviridae* (Supplementary Fig. S1). This suggests an evolutionary relationship between SRAV, the other SRAV-like viruses, and members of the family *Flaviviridae*. Nevertheless, the latest research on SRAV’s taxonomic classification places it within the family *Endornaviridae* (Postnikova et al. 2023). To elucidate the evolutionary status of SRAV, we started with an effort to reproduce the phylogenetic analysis of Postnikova *et al*. In short, the polyprotein sequences of SRAV, along with several representative members of the family *Flaviviridae* and family *Endornaviridae*, were retrieved and aligned. After trimming the aligned sequences, a maximum likelihood tree was constructed using the best-fit substitution model. The phylogenetic analysis revealed that SRAV clusters with the family *Flaviviridae*, supported by strong bootstrap values (Supplementary Fig. S3). However, this taxonomic classification contradicts the conclusions of most recent erroneous research about SRAV evolutionary status (Postnikova et al. 2023).

In summary, both the similarity of SRAV’s functional domains to those of flavivirids and the results of our phylogenetic analysis support the classification of SRAV with the family *Flaviviridae*, rather than *Endornaviridae*.

### 3.5 SRAV is evolutionary distant to the flavi-like viruses documented to infect plants

**Figure 4.**
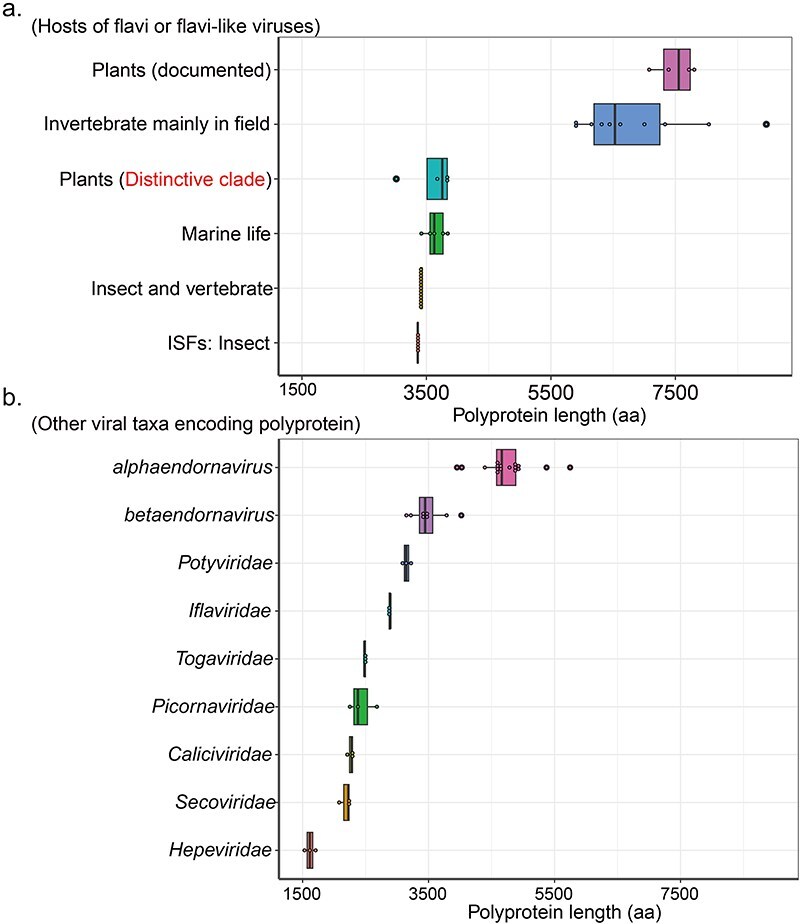
Comparison of polyprotein length of different viral groups. (a) Flavivirids or flavi-like viruses identified in different hosts. (b) Different virus families encoding large polyproteins.

Previous research has identified flavi-like viruses with atypical genome organization in plants, featuring genome lengths twice as long as those of classical flaviviruses (Fig. 4a). To explore this further, we first compared the polyprotein lengths of different flavivirids groups. We found that the previously identified plant flavi-like viruses have similar polyprotein lengths to flavi-like viruses found in plant-feeding invertebrates. However, the polyprotein of SRAV and the other novel SRAV-like viruses exhibited a length similar to that of classical flavivirids. This huge difference in polyprotein length suggests that SRAV may have a significant evolutionary distance from the plant viruses documented to infect plants. We also observed that SRAV, along with the two newly identified SRAV-like viruses, has a polyprotein length more similar to classical flaviviruses and other polyprotein-encoding viruses from different families (Fig. 4b).

**Figure 5.**
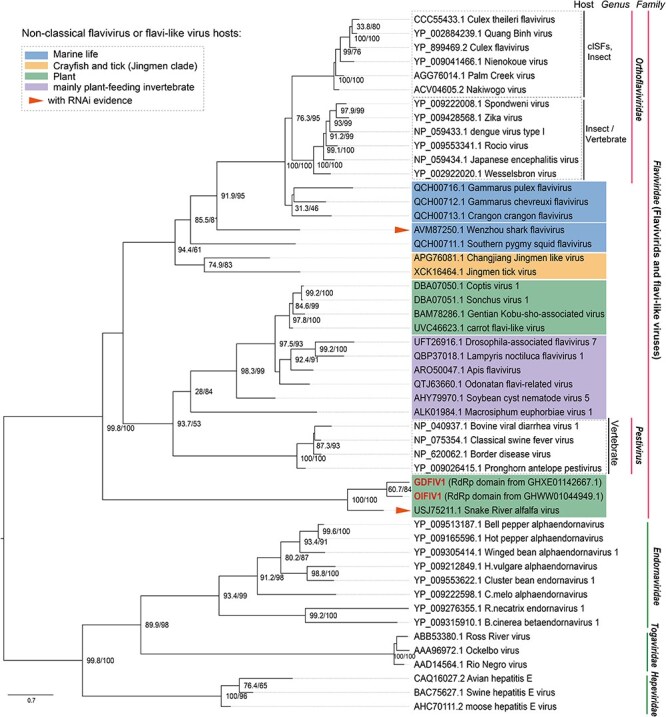
Maximum likelihood (ML) phylogenetic midpoint-rooted tree from the RdRp domain of SRAV representative members of family *Flaviviridae* and *Endornaviridae*, including those flavi-like viruses documented to infect plant (green area), marine life (blue area), crayfish and tick from Jingmen clade (yellow area), and plant-feeding invertebrates (purple area). The taxa represented by the red triangle are viruses that can activate the host’s RNAi antiviral response. Ultrafast bootstrap support value and SH-aLRT value are calculated and given at each node. The bar represents the number of substitutions per site (0.7).

To analyze the evolutionary differences between SRAV, other novel SRAV-like viruses, and currently published plant flavi-like viruses, a maximum likelihood phylogenetic tree was constructed based on the MSA of the RdRp domain region across different flavivirids groups and the representative members of a diverse of virus taxa. Notably, this evolutionary tree also incorporates the representatives of the Jingmen clade, which are currently classified by the International Committee on Taxonomy of Viruses as unclassified *Flaviviridae*. Additionally, we incorporated flavi-like viruses from nontraditional flavivirids hosts, such as marine organisms, invertebrates in the agricultural ecosystems, and plants, as well as some ssRNA viruses encoding large polyproteins from the families *Endornaviridae, Togaviridae*, and *Hepeviridae*. The phylogenetic tree reveals that flavi-like viruses documented to infect plants thus far are most closely related to flavi-like viruses primarily identified in plant-feeding invertebrates, forming a monophyletic clade. While reaffirming that SRAV is closely related to the family *Flaviviridae*, the evolutionary tree also highlights that SRAV and the known plant-infecting flavivirus-like viruses belong to distinct evolutionary clades. Furthermore, the evolutionary statuses of flavi-like viruses identified in marine life are consistent with the previous research (Parry et al. 2019) (Fig. 5).

## 4. Discussion

### 4.1 SRAV represents a *bona fide* virus that could trigger the host’s antiviral response in alfalfa

The comparison between the SRAV genome and alfalfa genomes using BLASTn reveals no significant sequence similarity, which suggests that SRAV may not be a host gene harboring an EVE. Additionally, a previous study found that some new clades of orphan viral genomes identified in fungi lack the classical catalytic triad motifs in the RdRp protein (Forgia et al. 2022). To determine whether SRAV and the other two SRAV-like viruses contains the complete RdRp motif and any nonclassical catalytic triads, we conducted a conserved motif analysis on the RdRp domain regions of these viruses. We found that, despite the RdRp protein of these viruses not showing detectable similarity to existing ones, except for SRAV itself, they contain conserved motifs A (D_X2-4_D), motif B (conserved G), and motif C (GDD), similar to those found in other eukaryotic viruses. This supports the idea that SRAV should represent a true virus (Fig. 1).

However, the best hits for two known viral domains of SRAV are the “Serine protease” of Japanese encephalitis virus (with a probability of 77.9% and an E-value of 6.3e^−5^) and the “RdRp catalytic core” of Classical swine fever virus (with a probability of 98.7% and an E-value of 1.3e^−12^), respectively. Both of these viruses are flavivirids belonging to the family *Flaviviridae*, which primarily infect invertebrates and vertebrates such as mosquitoes, zoonotic animals, and birds. Insects may introduce insect-specific viruses (ISVs) into plant tissues through feeding activities, which could lead to misleading conclusions when these ISV genomes are detected in plant transcriptomic datasets. Furthermore, SRAV could also be identified from plant-feeding thrips *Frankliniella occidentalis* (Dahan et al. 2022, Postnikova et al. 2023). Therefore, whether SRAV represent a real plant virus has become a new question.

Evolution has equipped plants with defense mechanisms to combat viral infections (Boualem et al. 2016). In brief, upon infection, RNA silencing is initiated by the recognition of viral dsRNAs or partially double-stranded hairpin RNAs. These viral RNAs are processed into vsiRNAs through the host’s small RNA biogenesis pathways (Boualem et al. 2016, Yang et al. 2018). Then we conducted a large-scale analysis of alfalfa small RNAs datasets to find evidence that SRAV could trigger the plant host’s antiviral response. We found that SRAV is detectable in various tissues and cultivars of alfalfa and exhibits a broad geographical distribution (Fig. 2b-e). The profile of SRAV-derived small interfering RNAs (vsiRNAs), including their length distribution and 5ʹ termini preference, displayed the typical characteristics of plant virus vsiRNAs (Figs 2, 3, Supplementary Fig. S2). It is crucial to highlight that the length distribution of SRAV vsiRNAs is primarily at 21 nt and 22 nt, with 21 nt being more abundant. This characteristic length distribution is conserved among plant virus vsiRNAs and is supported by the presence of similar patterns in other alfalfa viruses (such as Medicago sativa amalgavirus 1) identified in this study (Figs 2, 3, Fig. S2). However, due to the lack of publicly accessible small RNA datasets for thrips, we are unable to determine the vsiRNA patterns of potential thrips viruses for now. Nevertheless, given that SRAV is widely detected in various alfalfa sources and tissues (including roots, seeds, even embryo) (Figs 2, 3, Supplementary Table S2), it is highly unlikely that SRAV is a thrips virus introduced into alfalfa through feeding. Regarding the possibility that SRAV is a fungal virus, the widespread presence of SRAV in plants does not entirely rule out this possibility, as plants and endophytic fungi often exist in symbiosis. Notably, some studies have shown that plant viruses exhibit similar vsiRNA patterns when replicating in both fungi and plants (Pang et al. 2022, Wu et al. 2024). However, a deep examination of that research reveals that fungal viruses exhibit a strong preference for “U” at the 5ʹ termini of their vsiRNAs, a pattern not observed in plant viruses (Pang et al. 2022). In addition, we found that SRAV closely mirrors the characteristics of known alfalfa viruses (Medicago sativa alphapartitivirus 1, Medicago sativa alphapartitivirus 2, and Medicago sativa amalgavirus 1) (Figs 2, 3 and Supplementary Fig. S2). In addition, SRAV vsiRNAs account for a high proportion of total small RNAs, up to nearly 16% in a single sample (Fig. 3a). These findings support the conclusion that SRAV is a plant virus rather than a fungal virus.

### 4.2 The disagreement about the phylogenetic placement of SRAV

Having established that SRAV represents a real plant virus capable of eliciting host antiviral responses, we then investigate its evolutionary status. Determining the evolutionary status of orphan viral genomes remains challenging due to the absence of detectable sequence similarity to any known sequences. The main arguments of the article questioning SRAV’s evolutionary placement are based on several key points. Firstly, SRAV lacks the helicase domain typically found in the family *Flaviviridae* but does contain a poly(A) tail, which is not characteristic of family *Flaviviridae*. Additionally, SRAV is widely distributed in alfalfa, a biological characteristic more similar to viruses in the *Endornaviridae* family. The authors also constructed an evolutionary tree using Maximum Likelihood method using MEGA, which also showed that SRAV is “closer” to the *Endornaviridae* family. Furthermore, the authors conducted agarose gel electrophoresis and observed the presence of dsRNA of the approximately same size of SRAV (Postnikova et al. 2023). However, the arguments supporting these challenges are not sufficiently convincing. Since only one RdRp domain could be detected using CD-search or InterProScan in SRAV, an orphan viral genome without any detectable similarity to known sequences, it is highly possible that SRAV contains an unknown helicase domain rather than lacking a helicase domain entirely. As for containing a poly(A) tail, it could be explained by the polymorphism and diversity of the virus, especially for viruses identified across different host species. The observation of dsRNA bands in agarose gel electrophoresis is unsurprising, as single-stranded RNA viruses can also be detected as replicative intermediates (Roossinck et al. 2015). Moreover, some literature indicates that family *Endornaviridae* actually have a single-stranded genome (Valverde et al. 2019). However, the members of the family *Endornaviridae* was still documented to have a dsRNA genome structure in ICTV (https://ictv.global/report_9th/dsRNA/Endornaviridae).

To clarify SRAV’s phylogenetic status, we initially constructed a maximum likelihood tree using the same dataset as the study that questioned SRAV’s taxonomic classification, employing the best-fitted substitution model (Supplementary Fig. S3). To assess the robustness of these findings, we expanded the sequence datasets to include a broader range of virus taxa and reconstructed the phylogenetic tree (Fig. 5). Both analysis results consistently support the conclusion that SRAV is closely related to the family *Flaviviridae*, rather than the family *Endornaviridae*.

At this point, the debate regarding the evolutionary status of SRAV becomes clear. It is apparent that SRAV exhibits a closer evolutionary relationship to the family *Flaviviridae* than to the family *Endornaviridae*. Since the length of the bar below the phylogenetic tree represents the “site substitution rate,” the branch length in the tree can be used to infer the evolutionary distance between different taxa, as proposed by Yang (Yang and Cannatella 1998). From the phylogenetic tree (Figs 5, Supplementary S3), the branch length distance of the subclade composed by SRAV and other subclade is considerably long, indicating the substantial evolutionary distance between SRAV with other members of the family *Flaviviridae*. Based on our phylogenetic analysis, it is reasonable to classify SRAV as a distant member of the family *Flaviviridae* and name it a flavi-like virus. As SRAV genome is too divergent to detect similarity to any known sequence, it is conceivable to suggest that SRAV may ultimately serve as the founder of a new genus or even a higher taxonomic category within the broader hierarchy of flavi-like viruses (Dahan et al. 2022).

### 4.3 SRAV and SRAV-like viruses represent a distinct lineage of plant-hosted flavi-like viruses

In the research work in which SRAV was firstly identified in 2022, the authors proposed SRAV to be the first plant flavi-like viruses (Dahan et al. 2022). However, as early as 2013, a research work reported the identification of a plant flavi-like virus (Gentian Kobu-sho-associated virus GKaV) in gentian with unusual genome organization (Atsumi et al. 2013). Another novel plant flavi-like virus, carrot flavi-like virus 1, which shares its closest genetic similarity with GKaV, was discovered in populations of wild carrots (Schönegger et al. 2022). Subsequently, a research work introduced two novel plant flavi-like viruses by mining publicly available NCBI SRA datasets from Sonchus and Coptis samples (Debat and Bejerman 2023).

However, the genome length of the four documented plant flavi-like viruses with unusual genome organization is generally nearly twice as long as those of classic flaviviruses, and their polyprotein length is similar to that of flavi-like viruses identified primarily in plant-feeding invertebrates (Fig. 4). Furthermore, the phylogenetic tree illustrates that these four flavi-like viruses documented to infect plants are closely related to those flavi-like viruses primarily identified in plant-feeding invertebrates, forming a monophyletic clade (Fig. 5). Hence, the possibility that these four flavi-like viruses documented to infect plants are essentially flavi-like viruses of plant-feeding invertebrates introduced into plants through feeding or other behaviors, and subsequently detected accidentally in plant transcriptomic datasets, cannot be neglected. Additional evidence, such as infectious clones, virus inoculation experiments, or the induction of the host’s antiviral response, could provide further insights into whether these four viruses are indeed plant viruses. However, such evidence is currently unavailable for these four plant flavi-like viruses.

For comparison, SRAV exhibits a genome length similar to that of classical flavivirids (Fig. 4). While the SRAV genome diverges significantly, making it challenging to detect many known domains, the two domains identified by HHpred exhibit a genome organization similar to that of classic flaviviruses (Dahan et al. 2022). The phylogenetic analysis indicates that SRAV is evolutionary distant to the clade of plant flavi-like viruses with large genomes. Although we have questioned whether the four documented plant flavi-like viruses represent real plant viruses, we cannot deny that they are not plant viruses based solely on sequences. As a review paper has discussed, it seems possible that this group of plant flavi-like viruses were relatively recently transferred from invertebrates to plants and are undergoing active adaptation to the new host (Dolja et al. 2020).

Based on the results of functional domain annotation, genome organization, and phylogenetic analysis, we propose that SRAV and other two SRAV-like novel viruses represent a distinct lineage of plant-hosted flavi-like viruses. Furthermore, we provide evidence that plant flavi-like viruses could trigger the plant host’s antiviral response for the first time.

## Supplementary Material

veaf001_Supp

## Data Availability

Pipeline and scripts used in small RNA datasets processing and MSA files used to construct the phylogenetic tree can be accessed on the GitHub page: https://github.com/xuzhongtian/plant_flavi-like_viruses. The publicly available small RNA datasets analyzed in this study are from the BioProject as listed: SRP040470, SRP064230, SRP094823, SRP110842, SRP150034, SRP157110, SRP159567, SRP201465, SRP336109, and SRP434021.
