# Supplementary figures and images for: An orphan viral genome with unclear evolutionary status sheds light on a distinct lineage of flavi-like viruses infecting plants
Source: Virus Evol. 2025 Jan 3;11(1):veaf001. doi: 10.1093/ve/veaf001 (PMC11749231; doi:10.1093/ve/veaf001)

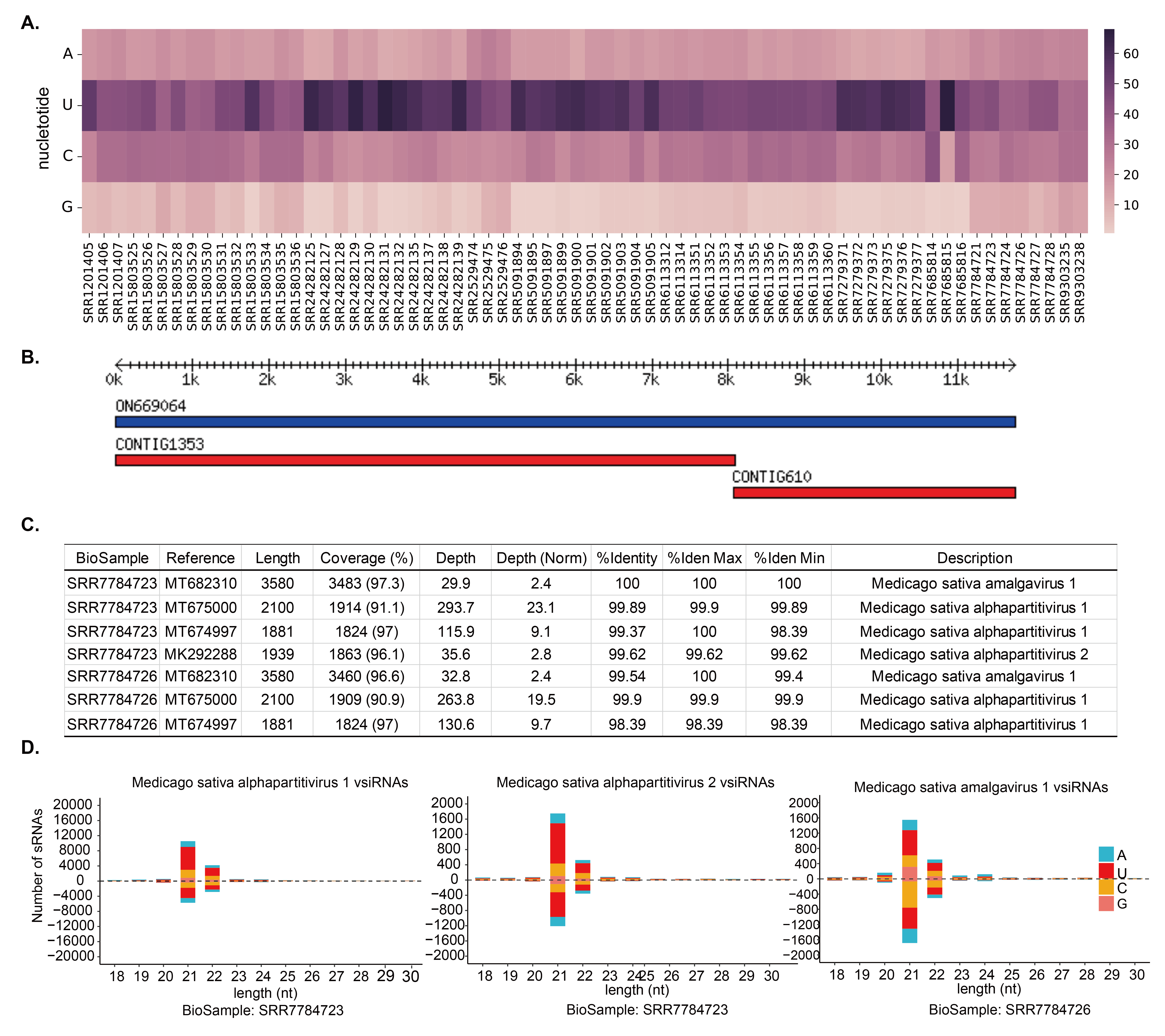

Supplement: veaf001_Supp [file veaf001_supp.zip › suppl_data/Fig. S2.tif]

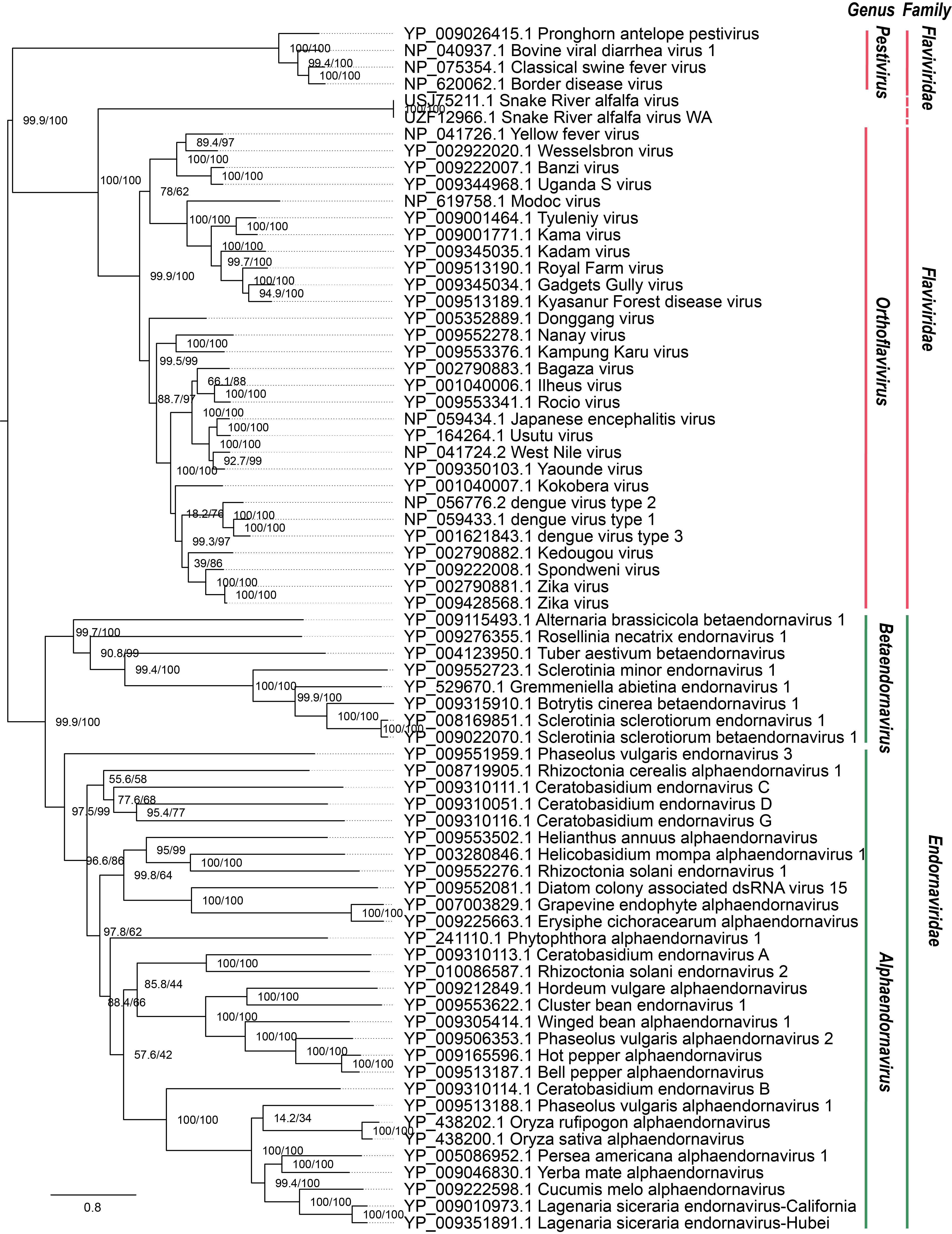

Supplement: veaf001_Supp [file veaf001_supp.zip › suppl_data/Fig. S3.tif]
